# Research on Deterioration Behavior of Magnesium Oxychloride Cement Under High Humidity and High Temperature

**DOI:** 10.3390/ma17215226

**Published:** 2024-10-26

**Authors:** Lingyun An, Ziyi Wang, Leichao Meng, Chenggong Chang, Zhifu Zhou, Fengyun Yan

**Affiliations:** 1 Qinghai Provincial Key Laboratory of Nanomaterials and Technology, Qinghai Minzu University, Xining 810007, China; anlingyun0825@126.com (L.A.); zy2534929264@126.com (Z.W.); leichao5166@163.com (L.M.); zhou2236366417@foxmail.com (Z.Z.); 2 Key Laboratory of Comprehensive and Highly Efficient Utilization of Salt Lake Resources, Qinghai Institute of Salt Lake, Chinese Academy of Sciences, Xining 810008, China; 3 Key Laboratory of Salt Lake Resources Chemistry of Qinghai Province, Xining 810008, China; 4 State Key Laboratory of Advanced Processing and Recycling of Non-Ferrous Metals, Lanzhou University of Technology, Lanzhou 730050, China

**Keywords:** magnesium oxychloride cement, deterioration behavior, high-humidity and high-temperature environment, microstructure, mechanical properties

## Abstract

To clarify the deterioration behavior of magnesium oxychloride cement (MOC) under conditions of high humidity and high temperature, we first placed MOC slurry samples in a simulated environment with a relative humidity of 97 ± 1% and a temperature of 38 ± 2 °C; then, we observed the changes in the macroscopic and microscopic morphology, water erosion depth, bulk density, phase composition, and mechanical properties of the samples. The results show that, over time, under the promotion of high temperature, water molecules infiltrate the MOC samples. This results in the appearance of cracks on the macroscopic surface of the MOC samples due to the volume expansion caused by the hydrolysis of P5 (5Mg(OH)_2_·MgCl_2_·8H_2_O) and the hydration of unreacted active MgO in the samples. The microscopic morphology of the samples changes from needle/gel-like, to flake-like, and finally leaf-like. Simultaneously, the major phase composition turns into Mg(OH)_2_. Since the structure of the samples becomes looser and the content of the main strength phase decreases, the overall compressive strength and flexural strength are both reduced. The compressive strength of the MOC slurry samples (0 day) is 93.2 Mpa, and the flexural strength is 16.4 MPa. However, after 18 days of treatment, water molecules reach the center of the MOC samples, and the MOC samples completely lose their integrity. As a result, their compressive and flexural strengths cannot be obtained.

## 1. Introduction

Climate change, one of the most prominent global environmental problems today, has been mainly caused by carbon dioxide (CO_2_) emissions. The chemical, steel, non-ferrous, and building material industries are the main sources of carbon emissions in the country [1]. To achieve the “double carbon” goal, vigorous research has been carried out [2], especially in the building material industry, leading to the development of low-carbon or negative-carbon building materials. For example, Grzegorz Ludwik Golewski [3] has reduced CO_2_ emissions through the use of a combination of fine waste fly ash particles and extremely fine particles of nano-silica in the structure of concrete.

Magnesium oxychloride cement (MOC), as a building material, was discovered based on the MgO-MgCl_2_-H_2_O system [4]. It is mainly composed of 5Mg(OH)_2_·MgCl_2_·8H_2_O (abbreviated as 5·1·8 phase, P5), 3Mg(OH)_2_·MgCl_2_·8H_2_O (abbreviated as 3·1·8 phase, P3), and Mg(OH)_2_. Notably, it possesses many advantages, such as good gelability, high early strength, high elasticity, low alkalinity, low thermal conductivity, fire resistance, and good wear resistance [5,6,7,8]. Its raw materials are mainly bischofite and light-burned magnesia. Bischofite is primarily obtained from the by-products generated during the extraction of potassium from the Qinghai salt lake, while light-burned magnesia is derived from the calcination of magnesite under a temperature of 750–850 °C. Interestingly, MOC can bond and solidify with a large variety of filling materials (e.g., sawdust, waste expanded polypropylene, and urban river sludge) [9,10,11]. Moreover, it has a low environmental impact throughout its life cycle and can absorb large amounts of CO_2_ from the air [12].

In view of the excellent performance of MOC and its low carbon footprint throughout its life cycle, considerable research has been conducted on the development of MOC’s raw materials, phase composition, microstructure, reaction mechanisms, kinetics, performance, degradation, and functional applications [13]. Especially, the water resistance and functional applications of MOC have been investigated in depth. For example, it has been found that MOC’s water resistance can be improved by adding hydroxyapatite [14], hydroxyacetic acid [15], or by creating a superhydrophobic surface [16,17], with the purpose of changing the morphology of MOC’s hydration products, stabilizing the P5 phase, inhibiting the formation of a large number of Mg(OH)_2_ crystals, increasing the density of the MOC structure, generating amorphous or other types of inorganic gels, and isolating water molecules. Additionally, by exploiting the excellent properties of MOC, new high-performance materials such as bone cement materials [18,19], photocatalytic composites [20,21], and functional materials [22] have been developed, therefore greatly expanding the application field of MOC.

The performance of MOC is closely related to factors such as the molar ratio, curing time, curing temperature, and environment. A. Timothy et al. [23] investigated the durability of MOC sheets in a high-humidity environment through accelerated experiments, and found that MOC sheets with different physical and chemical properties produced by different manufacturers have different levels of durability when exposed to high humidity. Ye and Feng et al. studied the effects of the curing temperature and maintenance temperatures on the mechanical properties of MOC, respectively. Their results showed that a curing temperature of 40 °C and a maintenance temperature of 40 °C are conducive to the development of MOC with good mechanical properties [24]. Still, no systematic research has been conducted on the outdoor durability of MOC in environments that are not only high in humidity but also high in temperature, which are characteristic of southern China.

To reveal the deterioration process of MOC in outdoor, high-humidity and high-temperature environments, we performed a continuous simulation of such conditions and determined the influence of the placement time on the apparent morphology, mechanical properties, and structure of MOC by analyzing its macroscopic morphology, water erosion depth, bulk density, and mechanical properties. Furthermore, we clarified the deterioration mechanism and durability of MOC under the selected conditions, providing a theoretical reference for the long-term application of MOC products in outdoor environments.

## 2. Materials and Methods

### 2.1. Principal Raw Materials

The main raw materials used in this study were bischofite from Golmud, China, and light-burned magnesia from Haicheng, China. Their chemical compositions are listed in Table 1 and Table 2.

### 2.2. Preparation of MOC Samples

Firstly, an MgCl_2_ aqueous solution with a mass fraction of 23.5% was prepared. Its specific preparation process was as follows: A total of 502.158 g of magnesium chloride hexahydrate (MgCl_2_·6H_2_O) was poured into a beaker. Then, 497.842 g of distilled water was added to the beaker. This was stirred for 5 min at room temperature to fully dissolve the magnesium chloride hexahydrate. Eventually, a 23.5% magnesium chloride solution with a mass fraction of 23.5% was formed.

MOC samples with a molar ratio of active MgO, MgCl_2_, and H_2_O of 7.2:1:16 were obtained. Light-burned magnesium oxide powder and the pre-prepared 23.5 wt.% MgCl_2_ aqueous solution were mixed and stirred evenly to form a slurry, which was then injected into a 40 mm × 40 mm × 160 mm rectangular mold and naturally hardened in an indoor environment (relative humidity = 40 ± 1%, temperature = 21 ± 1 °C). After 24 h, the hardened pastes were removed from the mold and then continuously cured in an indoor environment for 28 days. These samples were the MOC slurry samples and they were labeled as 0 day.

### 2.3. High-Humidity and High-Temperature Experiment

**Figure 1:**
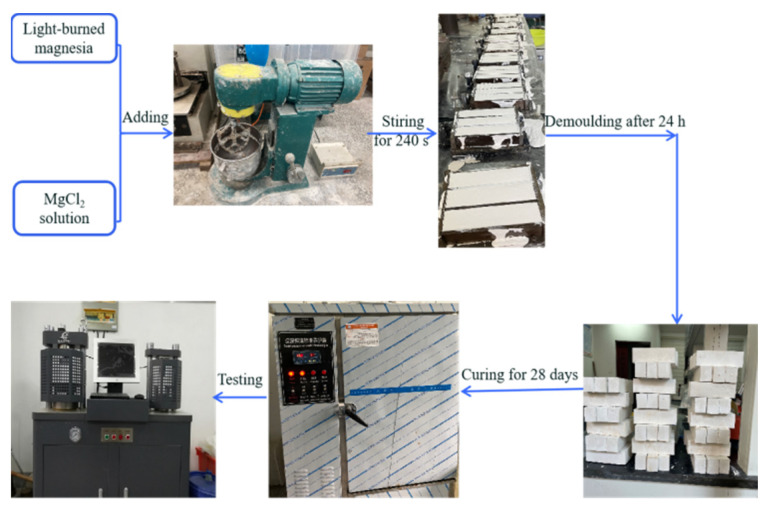
Schematic diagram of the experimental process.

The MOC slurry samples (0 day) were placed in a maintenance box, the relative humidity and temperature of which were set to 97 ± 1% and 38 ± 2 °C at atmospheric pressure, respectively. After 4, 10, 14, and 18 days, the samples were taken out of the box (Figure 1) for further characterization.

### 2.4. Characterization

The quality of the samples was tested by using an electronic balance (SPX622ZH, OHAUS, Shanghai, China). For each placement time, six samples were taken, and each sample was measured five times. The average value was indicated with “M” (unit of measure:g). The weight (g/cm^3^) was calculated according to Equation (1):

Bulk density = M/256(1)

where 256 corresponds to the sample volume (in cm^3^).

**Figure 2:**
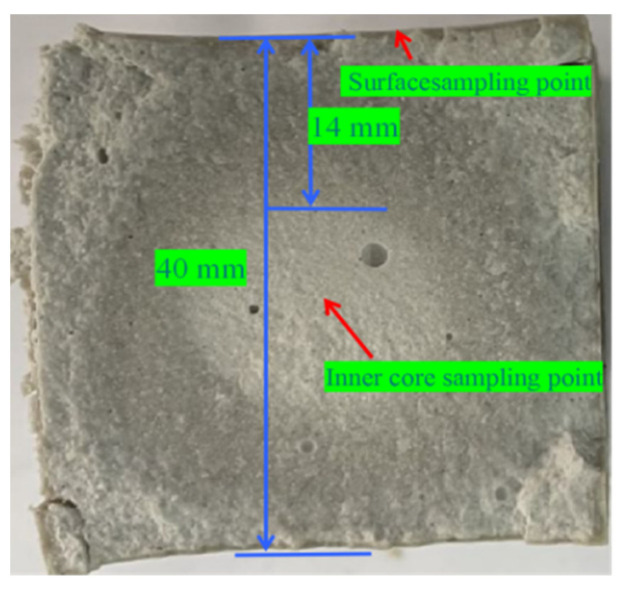
Measurement of the water erosion depth and sampling points.

The microstructure, phase composition, and thermal stability of MOC were analyzed by using a field emission scanning electron microscope (FE-SEM, model SU8010, Hitachi High-Tech, Tokyo, Japan), an X-ray diffraction meter (XRD, model D8 Discover, Bruker, Karlsruhe, Germany), a Fourier-transform infrared spectrum (FTIR, model Thermo-Nicolet Nexus, Madison, New York, USA), and a thermogravimetric analysis/differential scanning calorimeter (TG−DSC, model STA449F3, Selb, Bavaria, Germany). The compressive and flexural strengths of the MOC samples were tested in accordance with GB/T17671-2020 of the “Cement Rubber Sand Strength Test Method” (ISO method) by using a micro-electromechanical electro-hydraulic servo pressure testing machine (model HYE-300B-D, Beijing sanyuweiye testing machine Co., Ltd, Beijing, China). Then, a vernier caliper was used to measure the water erosion depth in the cross-section of the sample, and the sampling point was marked (Figure 2): the water erosion depth of the sample after 14 days was found to be 14 mm.

## 3. Results

### 3.1. Macro-Morphological Analyses

**Figure 3:**
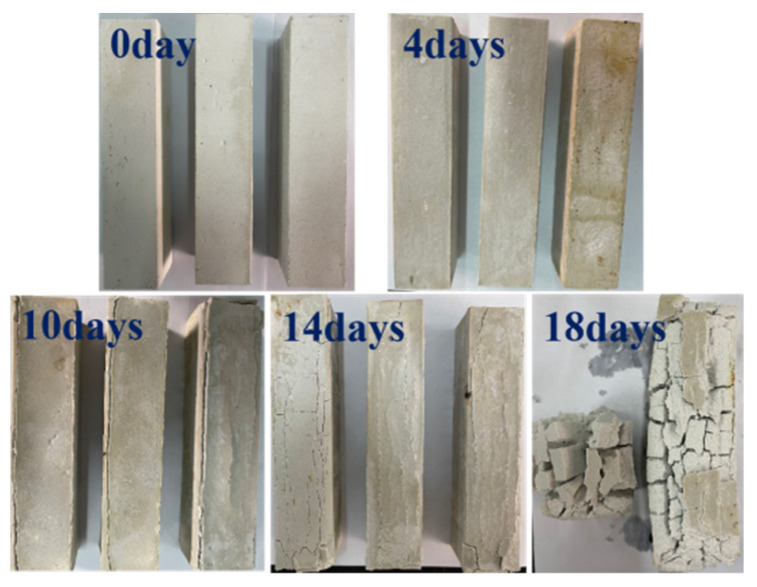
Macro photos of MOC samples placed in a high-humidity and high-temperature environment for different times.

Figure 3 shows some macroscopic photos of the MOC samples, which have been subjected to high-humidity and high-temperature conditions for different time intervals. The surface of the MOC slurry sample (0 day) is milky white, complete, and dense, without micro-cracks. When the MOC slurry sample is placed in a high-humidity and high-temperature environment for 4 days, its surface becomes dark, but it is still intact, with no obvious micro-cracks. After being placed for 10 days under high-humidity and high-temperature conditions, the surface of the MOC sample exhibits some cracks, especially at its edges. This is mainly attributed to volume expansion caused by phase transformation and its heterogeneity. The surface of the MOC sample after 14 days of treatment shows a large number of cracks, and the edges and corners show signs of spalling. Further prolonging the placement time to 18 days, the surface of the MOC sample shows even more and wider cracks and has completely lost its integrity. This shows that water molecules gradually infiltrate the MOC samples over time, gradually deteriorating their surfaces on a macroscopic scale.

### 3.2. Depth of Water Erosion and Bulk Density Analyses

**Figure 4:**
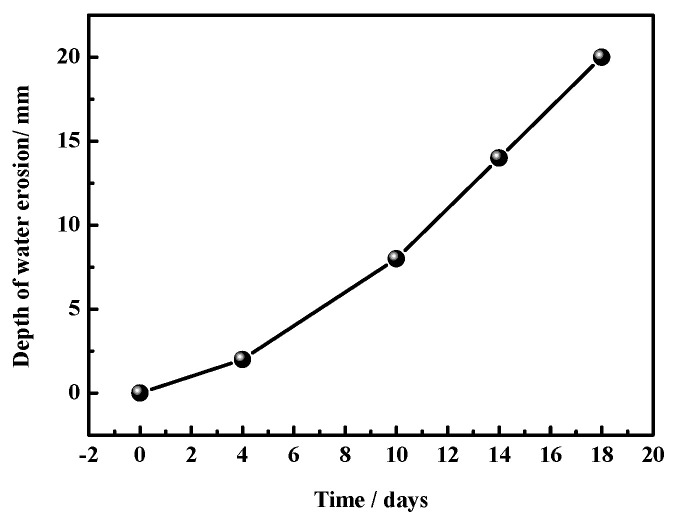
Water erosion depths of MOC samples placed in high-humidity and high-temperature conditions for different times.

**Figure 5:**
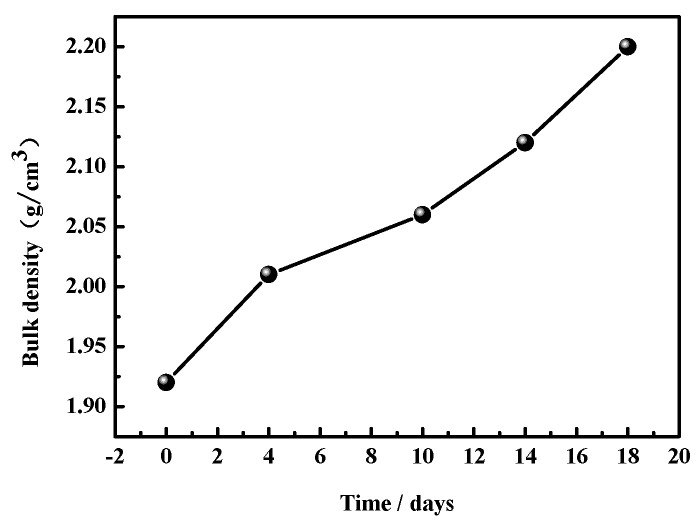
Bulk densities of MOC samples placed in high-humidity and high-temperature conditions for different times.

Figure 4 and Figure 5 display the water erosion depths and the bulk densities of the MOC samples, respectively. In particular, Figure 4 shows how the depth of water erosion gradually increases over time under the test conditions. After 18 days of placement, the water erosion depth is 20 mm, indicating that water molecules have reached the center of the sample. Figure 5 shows that the bulk density gradually increases as the placement time goes on. This result can be explained by the continuous infiltration of water molecules into the samples during the experiment.

### 3.3. Mechanics Properties Analyses

**Figure 6:**
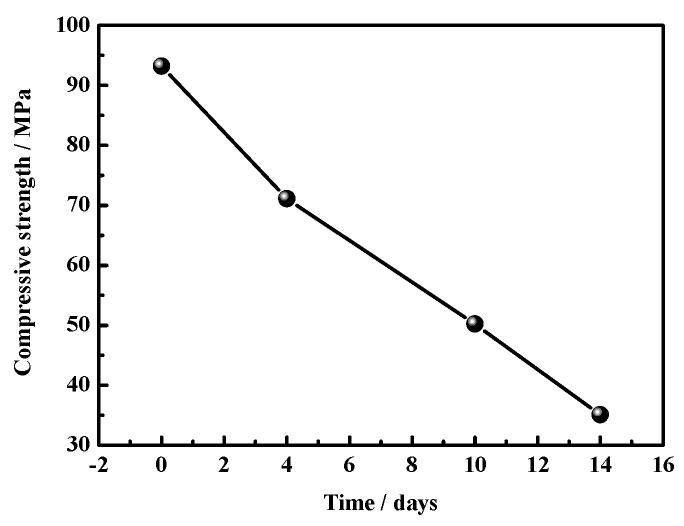
Compressive strengths of MOC samples placed in high-humidity and high-temperature conditions for different times.

**Figure 7:**
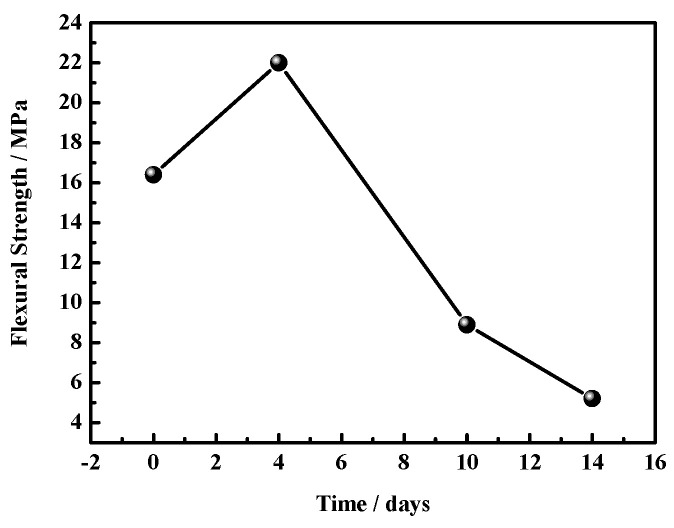
Flexural strengths of MOC samples placed in a high-humidity and high-temperature environment for different times.

Figure 6 and Figure 7 present the compressive and flexural strengths of the MOC specimens. Figure 6 shows how, over time, compressive strength gradually decreases, while the magnitude of the decline increases. The MOC sample placed in a high-humidity and high-temperature environment for 4 days has a compressive strength of 71.1 MPa. After 14 days, its compressive strength is 36.1 MPa. However, after 18 days, the compressive strength of the MOC sample cannot be obtained due to it being broken. Moreover, Figure 7 shows that the flexural strength of the MOC sample treated for 4 days is the highest: 22 MPa. This indicates that the MOC sample still possesses excellent mechanical properties after 4 days of treatment. As the placement time further increases, the flexural strength decreases. When the placement time is 14 days, the flexural strength of the MOC sample is 5.2 MPa. However, after 18 days, its flexural strength cannot be obtained.

### 3.4. XRD Analyses

**Figure 8:**
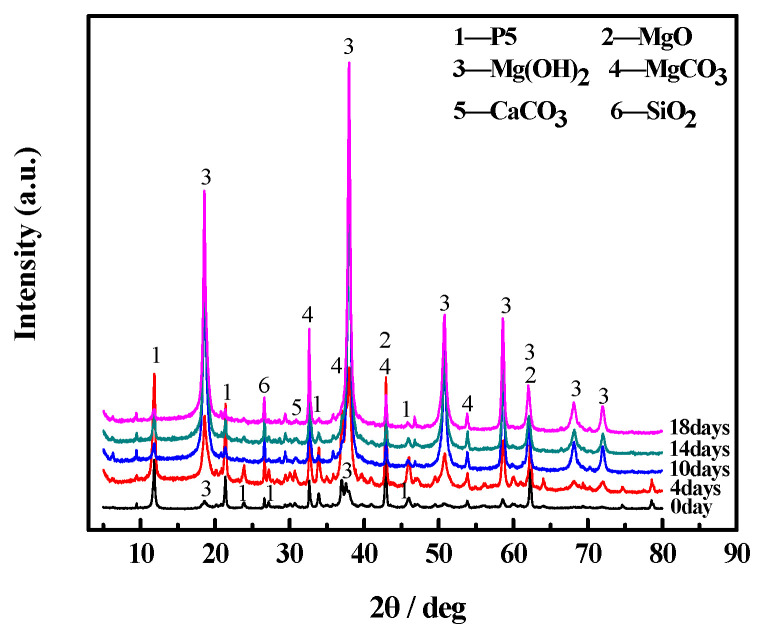
XRD patterns of the surface layers of MOC samples placed in a high-humidity and high-temperature environment for different times.

**Figure 9:**
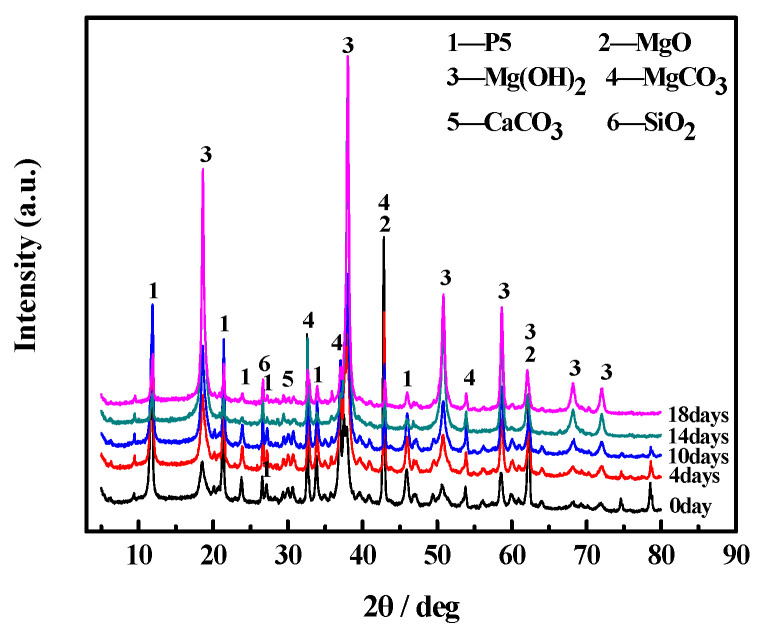
XRD patterns of the inner cores of samples placed in a high-humidity and high-temperature environment for different times.

Figure 8 and Figure 9 show the XRD patterns of the surfaces and inner cores of the MOC samples, respectively. All the samples contain the MgCO_3_, CaCO_3_, and SiO_2_ phases, which derive from magnesite. Meanwhile, the MgO shown in the XRD patterns originates from light-burned MgO [25].

The P5 (5Mg(OH)_2_·MgCl_2_·8H_2_O) diffraction peaks of the surface layers of the initial MOC slurry sample (0 day) and the sample treated for 4 days do not differ much. However, the Mg(OH)_2_ diffraction peak of the sample after 4 days of placement is higher and the MgO diffraction peak is lower compared to those of the initial MOC slurry sample. This suggests that the content of Mg(OH)_2_ in the MOC sample after suffering from 4 days of placement is higher than that in the initial MOC slurry sample, while the content of MgO is lower. The XRD patterns of the samples after 10 days, 14 days, and 18 days of treatment are similar. However, their P5 diffraction peaks are much lower than that of the initial MOC slurry sample, while their Mg(OH)_2_ content is higher and their MgO content is almost null (Figure 8).

No significant changes can be observed in the diffraction peaks of P5, Mg(OH)_2_, and MgO among the samples placed for 0 days, 4 days, 10 days, and 14 days, respectively. However, the Mg(OH)_2_ diffraction peak of the sample treated for 18 days is obviously higher than that of the other samples, but the diffraction peak of P5 is significantly lower, while the MgO diffraction peak is almost non-existent. This indicates that over time, under the tested environmental conditions, the Mg(OH)_2_ content of the surfaces and inner cores of the samples gradually increases, while the P5 content continuously decreases. After 18 days of placement, MgO almost completely disappears from the MOC (Figure 9).

### 3.5. TG-DSC Analyses

**Figure 10:**
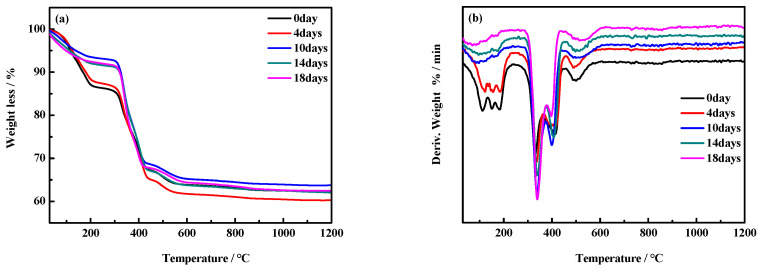
TG curves (**a**) and DTG curves (**b**) of the surface layers of MOC samples placed in a high-humidity and high-temperature environment for different times.

Figure 10 illustrates the thermogravimetry (TG) and derivative thermogravimetry (DTG) curves of the MOC samples’ surface layers. The TG curves of the MOC slurry sample (0 day) and the sample treated for 4 days indicate that their total weight loss is 22.81% in the temperature range of 0–350 °C. This can be attributed to the loss of water molecules from 5Mg(OH)_2_·MgCl_2_·8H_2_O. In addition, the DTG curves of the same samples show endothermic peaks at 111 °C, 150 °C, and 180 °C, indicating that a total of eight water molecules should have been gradually removed from 5Mg(OH)_2_·MgCl_2_·8H_2_O. The endothermic peaks observed at 330 °C, 400 °C, and 500 °C should correspond to enhanced decomposition peaks of Mg(OH)_2_ and P5 [26,27]. The TG curves of MOC samples placed for 10 days, 14 days, and 18 days indicate that there should have been a weight loss of 8% between 0–350 °C, possibly due to the decomposition of P5. At the same time, the DTG curves of MOC samples after 10 days, 14 days, and 18 days of placement show significantly smaller endothermic peaks at 111 °C, 150 °C, and 180 °C compared to the MOC slurry sample (0 day) as well as the sample treated for 4 days, indicating a lower content of P5. Meanwhile, the peak at 330 °C is significantly greater, suggesting that the Mg(OH)_2_ content is higher. These observations are consistent with the results of XRD surface analyses.

**Figure 11:**
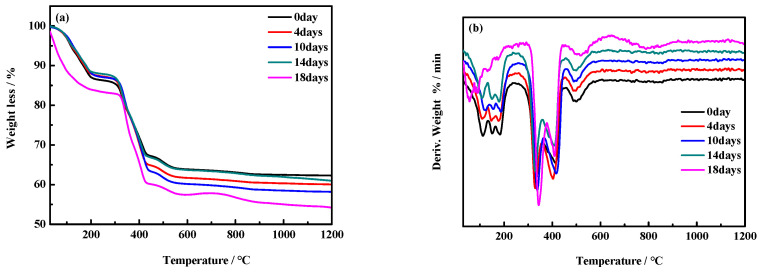
TG curves (**a**) and DTG curves (**b**) of the inner cores of MOC samples placed in a high-humidity and high-temperature environment for different times.

Figure 11 shows the TG and DTG curves of the MOC samples’ inner cores. In particular, Figure 11a indicates that the total weight loss of the samples treated for 0 days, 4 days, 10 days, and 14 days is approximately 22% between 0–350 °C. Considering the endothermic peaks at 111 °C, 150 °C, and 180 °C in the DTG curves, this weight loss may have been induced by the decomposition of P5. Meanwhile, the endothermic peaks at 330 °C, 400 °C, and 500 °C are attributed to the decomposition of P5 and Mg(OH)_2_ (Figure 11b). This is consistent with the TG and DTG analysis results of the surface layers of the MOC slurry sample (0 day) and the sample treated for 4 days: under the tested environmental conditions, water molecules do not completely infiltrate the inner cores of the MOC samples after 14 days. The TG and DTG curves of the inner core of the sample treated for 18 days exhibit changes similar to those of the surface layers of the samples after 10 days, 14 days, and 18 days of treatment (Figure 10). The intensity of the endothermic peaks at 111 °C, 150 °C, and 180 °C in the DTG curves is significantly lower, and the peak at 330 °C is evidently higher, indicating a higher content of Mg(OH)_2_. This suggests that water molecules completely infiltrate the cores of the samples after 18 days under the tested environmental conditions.

### 3.6. FTIR Spectrum Analyses

**Figure 12:**
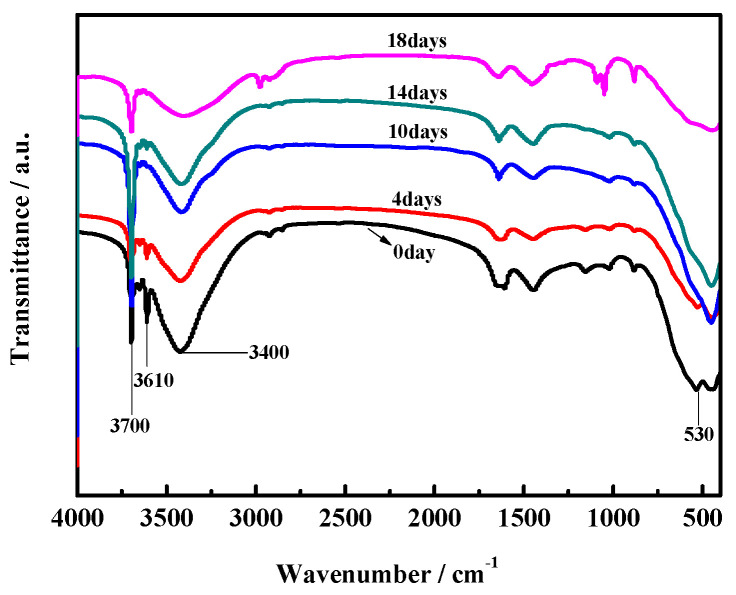
FTIR spectra of the surface layers of MOC samples placed in a high-humidity and high-temperature environment for different times.

**Figure 13:**
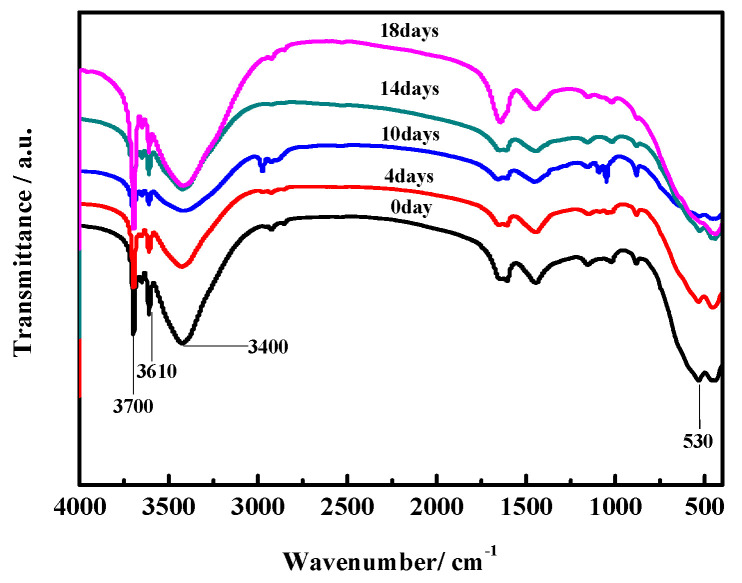
FT-IR spectra of the inner cores of MOC samples placed in high-humidity and high-temperature environment for different time.

Figure 12 and Figure 13 show the FTIR spectra of the surface layers and inner cores of the MOC samples. In previous studies [28], it has been noted that the stretching vibration absorption band of the Mg–O bond is located at 530 cm^−1^ (i.e., the characteristic peak of MgO). The characteristic peak of the coordination water molecular vibration is located between 3330–3500 cm^−1^ instead. The absorption sharpness of 3670 cm^−1^ and 3610 cm^−1^ depends on the non-aqueous hydroxyl (i.e., −OH) of the telescopic vibration peak superimposed onto crystalline water, which belongs to the characteristic peak of P5. The peak intensity at 3700 cm^−1^ is different for all samples, indicating that the content of Mg(OH)_2_ varies among them. The MOC slurry sample (0 day) and the sample treated for 4 days mainly contain the characteristic peaks of P5 and MgO. However, for the samples placed for 10 days, 14 days, and 18 days, the characteristic peak of MgO is not visible and the characteristic peak of P5 is obviously lower. Meanwhile, the characteristic peak of Mg(OH)_2_ gradually becomes higher when MOC samples are treated from 10 days to 18 days (Figure 12). This shows that over time, under high-humidity and high-temperature conditions, water molecules continuously infiltrate the MOC samples, resulting in the disappearance of all MgO from their surface, a gradual decrease in P5 content, and a significant increase in Mg(OH)_2_ content. These findings are in line with the XRD and TG-DSC analysis results.

Figure 13 shows that the characteristic peaks of P5 and MgO for the inner cores of MOC samples placed separately in a high-humidity and high-temperature environment for 0 days, 4 days, 10 days, and 14 days are quite similar to one another. However, for the MOC sample treated for 18 days, the characteristic peak of P5 is lower, that of MgO is not visible, and that of Mg(OH)_2_ is significantly higher. Combining this information with that shown in Figure 12, it can be inferred that the innermost cores of the MOC samples are damaged at a much slower rate due to the protection provided by the outer layers. After 18 days of treatment, eventually, the inner cores of the samples also undergo deterioration. Additionally, the content of P5 diminishes, MgO disappear, and the Mg(OH)_2_ content increases, as also indicated by the results of XRD and TG-DSC analyses.

### 3.7. SEM Analyses

**Figure 14:**
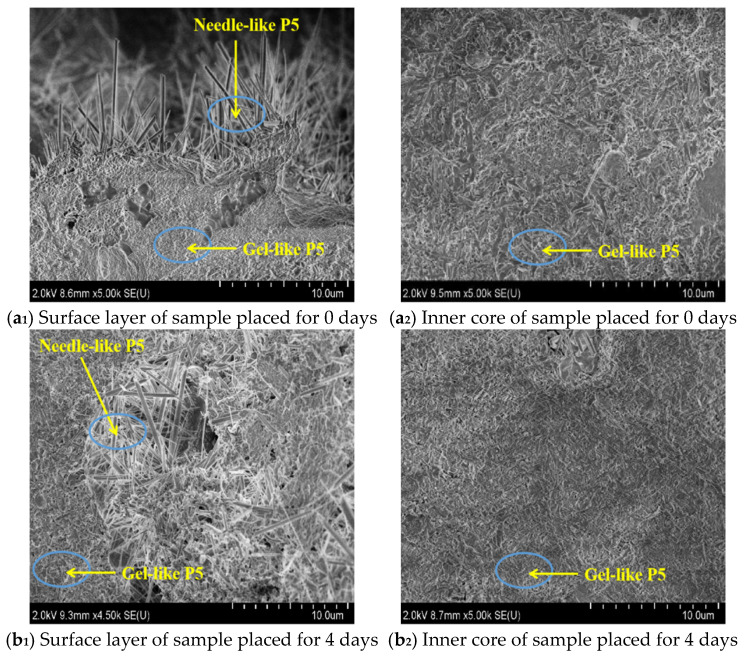

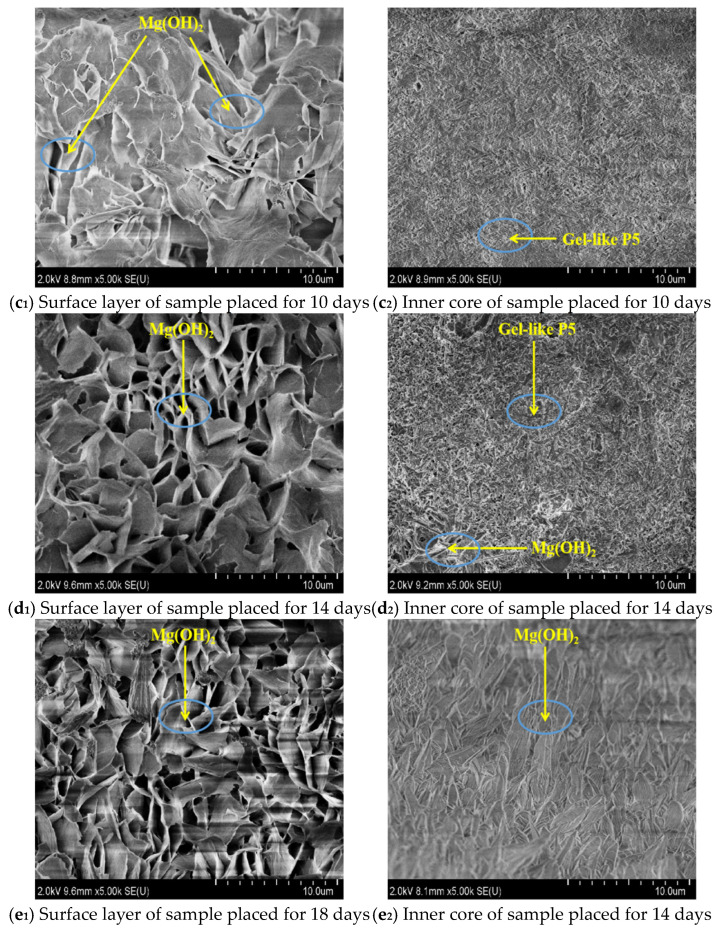
SEM micrographs showing the morphologies of the microscopic surfaces (**a_1_**–**e_1_**) and inner cores (**a_2_**–**e_2_**) of the samples placed in a high-humidity and high-temperature environment for different times.

Figure 14 shows the microscopic morphologies of the surface and inner core of MOC samples. Those of the MOC slurry sample (0 day) are needle-like and gel-like. According to the literature [29,30] and the results of XRD as well as FTIR, they may be P5 (5Mg(OH)_2_·MgCl_2_·8H_2_O). They occur alternately, resulting in a relatively dense structure. The morphologies of the MOC sample placed in a high-humidity and high-temperature environment for 4 days are not significantly different from those of the MOC slurry sample. The surface layer of the sample after 10 days of placement exhibits a flake-like morphology that may belong to Mg(OH)_2_, in keeping with the literature [30] and the analysis of XRD as well as FTIR. When the MOC sample is in a high-humidity and high-temperature environment for 14 days, it shows a surface layer with an obvious leaf-like morphology, while its inner core exhibits a slight leaf-like morphology. Further prolonging the placement time to 18 days, the MOC sample displays a surface layer and an inner core with leaf-like morphologies, while its structure is loose and porous. This suggests that over time, under the tested environmental conditions, water molecules gradually infiltrate the MOC samples, altering their micromorphology: the MOC sample after 18 days of treatment, the surface layer of which is in contact with the water molecules for the longest time, is the most obviously deteriorated.

## 4. Discussion

MOC is an air-hardening cementitious material, the mechanical properties of which are primarily determined by a series of factors including macroscopic and microscopic structures, phase composition, and compactness. The MOC slurry sample that has not been exposed to outdoor, high-humidity and high-temperature environments, exhibits a milky white color. This sample has no cracks on its surface and possesses a complete macrostructure. Its microscopic morphology is mostly and alternately needle/rod-like and gel-like, resulting in a relatively dense structure. Additionally, this sample is mainly composed of P5, Mg(OH)_2_, and MgO phases. Therefore, it has good mechanical properties: a compressive strength of 93.2 MPa and a flexural strength of 16.4 MPa.

When the MOC slurry sample is placed in a dry and normal temperature environment (relative humidity 15 ± 1%, temperature 25 ± 2 °C), its compressive strength and flexural strength gradually decrease with the extension of the placement time. After 18 days, the compressive strength of the MOC sample is 80.9 MPa and its flexural strength is 12.5 MPa, declines of 12.3 MPa and 3.9 MPa, respectively, in comparison with those measurements of the MOC slurry sample, as shown in Table 3 and Table 4. This may be attributed to the volatilization of free water present in the MOC sample. The research [31] has shown that a small quantity of water molecules can favor the connection between P5 gels, and also between P5 gels and other phases, thereby enhancing the compressive and flexural strengths of MOC samples. Therefore, the volatilization of free water in a dry environment will lead to the decline of the compressive and flexural strengths of the MOC sample.

Compared with a dry and normal temperature environment (relative humidity 15 ± 1%, temperature 25 ± 2 °C), when the MOC slurry sample is applied in a dry but high-temperature environment (relative humidity 15 ± 1%, temperature 38 ± 2 °C), the high temperature slows down the decline of the compressive strength of the MOC sample. As a result, the compressive strength of the MOC sample placed for 18 days only decreases by 0.3 MPa compared to the MOC slurry sample, remaining almost unchanged. However, the high temperature promotes a slight decrease in flexural strength. When the placement time is extended to 18 days, the flexural strength reduces to 11.1 MPa.

Meanwhile, compared to a dry and normal temperature environment (relative humidity 15 ± 1%, temperature 25 ± 2 °C), when the MOC slurry sample is in a normal temperature but high-humidity environment (temperature 25 ± 2 °C, relative humidity 97 ± 1%), the high-humidity environment significantly aggravates the decline in the compressive and flexural strengths of the MOC sample, and promotes the deterioration of the mechanical properties of the MOC sample, as shown in Table 3 and Table 4. The high-humidity environment provides more water molecules, and MOC is a heterogeneous body composed of MgCO_3_, CaCO_3_, SiO_2_, P5, Mg(OH)_2_, and active MgO. As the placement time extends, water molecules gradually infiltrate the sample and react with active MgO, generating Mg(OH)_2_ (Equation (2)) [32]. In addition, part of the P5 is also decomposed into Mg(OH)_2_ (Equation (3)). Therefore, when the MOC slurry sample is applied in a normal temperature but high-humidity environment, the high humidity causes the MOC sample to come into contact with more water molecules. As a result, with the extension of the placement time, the compressive and flexural strengths of the MOC slurry sample decrease significantly, as shown in Table 3 and Table 4.

active MgO + H_2_O→Mg(OH)_2_ (2)

5Mg(OH)_2_·MgCl_2_·8H_2_O + H_2_O →5Mg(OH)_2_ + MgCl_2_ (loss) (3)

However, when the MOC slurry sample is placed in a high-humidity and high-temperature environment (temperature 38 ± 2 °C, relative humidity 97 ± 1%), the high-humidity environment provides more water molecules. Moreover, a temperature of 40 °C stimulates the Brownian motion of these water molecules (the higher the temperature, the more vigorous this motion would be), increasing the diffusion speed of these water molecules, so that more water molecules can enter the sample at a faster speed than at normal temperature, influencing the macroscopic performance and microstructure of the MOC sample. According to the above research results, the MOC sample placed for 4 days is infiltrated by water molecules (erosion depth = 2 mm). As a result, its surface turns dark, and its bulk density increases from 1.92 g/cm^3^ to 2.01 g/cm^3^; still, no cracks are visible on its surface. The intrusion of water molecules leads to a decrease in the content of P5 (i.e., the main strength phase), which inevitably results in a decrease in compressive strength. However, due to the intrusion of a small amount of water, the flexural strength of the MOC sample placed for 4 days reaches the highest value, 22.0 MPa.

**Figure 15:**
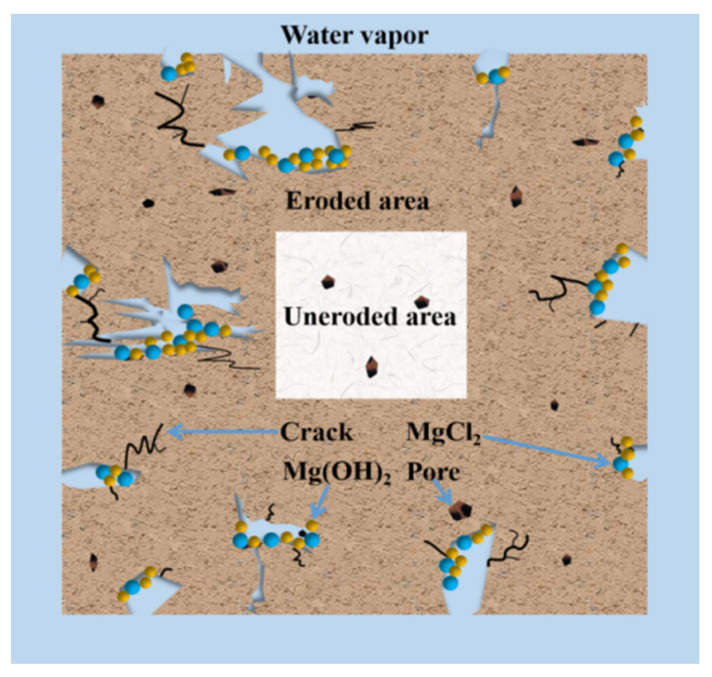
Schematic picture of working principle of MOC sample under high-temperature and high-humidity.

With respect to the MOC sample which is placed for 10 days under a relative humidity of 97 ± 1% and a temperature of 38 ± 2 °C, because of the increase in the amount of water molecules entering the MOC sample, more P5 and the unreacted active MgO undergo hydrolysis and hydration reactions, generating more Mg(OH)_2_. Since the volume of Mg(OH)_2_ is larger than that of MgO, it causes the volume expansion of the MOC sample. As a result, there are cracks appearing at the edge of the sample that is continuously treated for 10 days, as shown in Figure 3. In addition, MgCl_2_ generated by the decomposition of P5 is highly soluble in water, which leads to the formation of a micro-sheet structure: similar to karst landforms. In summary, the MOC sample that has been continuously placed for 10 days, exhibits macroscopic cracks on its surface layer; moreover, its microstructure is flaky and porous, and its main strength phase (P5) content is lower compared to that of previous samples. Hence, its compressive and flexural strengths are relatively low. The possible deterioration mechanism of the MOC sample in outdoor, high-humidity and high-temperature environments is illustrated in Figure 15.

The MOC sample placed for 14 days under a temperature of 38 ± 2 °C and relative humidity of 97 ± 1% displays an erosion depth of 14 mm. This results in a large number of macroscopic cracks on its surface, and the partial exfoliation of its edges and corners. The microscopic morphology of the surface layer of this sample is similar to that of the sample treated for 10 days, the karst landform is more obvious, and the sample is mostly composed of Mg(OH)_2_. Furthermore, the core of the sample after 14 days of placement exhibits a hint of flake morphology, which results in relatively low compressive strength (35.1 MPa) and flexural strength (5.2 MPa).

The MOC sample which is treated for 18 days has completely lost its integrity, and a large number of variously shaped and long cracks occur on its surface. The depth of water erosion is 20 mm, indicating that erosion reaches the core of the sample: the microscopic morphology of its inner core is leaf-like. Moreover, this sample is mainly composed of Mg(OH)_2_ and its mechanical properties cannot be obtained.

## 5. Conclusions

(1) The MOC obtained from the hardening reaction of MgO and MgCl_2_ solutions is a heterogeneous material with a low carbon footprint. The MOC slurry is mainly composed of P5, Mg(OH)_2_, and unhydrated MgO. Its microscopic morphology is needle/rod-like and gel-like, and its structure is relatively dense. Therefore, it has excellent mechanical properties, with a compressive strength of 93.2 MPa and a flexural strength of 16.4 MPa.

(2) A high-humidity and high-temperature environment exhibits a significant influence on MOC samples. In such an environment, over time, water molecules gradually infiltrate the MOC samples. This process is promoted by high temperature, making P5 and the unreacted active MgO undergo hydrolysis and hydration reactions. Therefore, the surface color of MOC samples changes from milky white to dark; furthermore, micro-cracks form and gradually widen, the microscopic morphology of the surface layer changes from needle-like and gel-like to flake-like and leaf-like, and the predominant phases gradually change from MgO and P5 to Mg(OH)_2_. After 18 days of treatment, the microscopic morphology of both the surfaces and cores of the MOC samples becomes leaf-like; furthermore, the predominant phase is Mg(OH)_2_. At this point, MOC has completely lost its integrity, resulting in its compressive strength and flexural strength values being unobtainable.

(3) From these findings, we infer that MOC needs to be improved in some way (internally or externally) to improve its durability in outdoor high-humidity and high-temperature environments.

## Figures and Tables

**Table 1:** Chemical composition of bischofite.

Composition	MgCl_2_	NaCl	MgSO_4_	KCl	CaCl_2_	Water-Insoluble Matter
Content (wt.%)	44.90	0.13	0.06	0.01	0.03	0.27

**Table 2:** Chemical composition of light-burned magnesia.

Composition	MgO	MgCO_3_	CaCO_3_	f-CaO	Acid-Insoluble Matter
Content (wt.%)	69.52	19.80	1.34	0.38	8.41

**Table 3:** Compressive strength (MPa) of MOC samples in different environments.

Environments	0 Day	4 Days	10 Days	14 Days	18 Days
Relative humidity = 15 ± 1%, temperature = 25 ± 2 °C	93.2	85.3	84.1	80.3	80.9
Relative humidity = 15 ± 1%, temperature = 38 ± 2 °C	93.2	89.3	91.9	91.1	92.9
Relative humidity = 97 ± 1%, temperature = 25 ± 2 °C	93.2	63.2	51.6	47.5	32.2
Relative humidity = 97 ± 1%, temperature = 38 ± 2 °C	93.2	71.1	50.2	35.1	0.0

**Table 4:** Flexural strength (MPa) of MOC samples in different environments.

Environments	0 Day	4 Days	10 Days	14 Days	18 Days
Relative humidity = 15 ± 1%, temperature = 25 ± 2 °C	16.4	14.3	14.4	13.9	12.5
Relative humidity = 15 ± 1%, temperature = 38 ± 2 °C	16.4	9.9	10.5	10.8	11.1
Relative humidity = 97 ± 1%, temperature = 25 ± 2 °C	16.4	13.0	11.6	8.9	2.3
Relative humidity = 97 ± 1%, temperature = 38 ± 2 °C	16.4	22.0	8.9	5.2	0.0

## Data Availability

The original contributions presented in the study are included in the article, further inquiries can be directed to the corresponding authors.
